# Nomenclature for renal replacement therapy in acute kidney injury: basic principles

**DOI:** 10.1186/s13054-016-1489-9

**Published:** 2016-10-10

**Authors:** Mauro Neri, Gianluca Villa, Francesco Garzotto, Sean Bagshaw, Rinaldo Bellomo, Jorge Cerda, Fiorenza Ferrari, Silvia Guggia, Michael Joannidis, John Kellum, Jeong Chul Kim, Ravindra L. Mehta, Zaccaria Ricci, Alberto Trevisani, Silvio Marafon, William R. Clark, Jean-Louis Vincent, Claudio Ronco, on behalf of the Nomenclature Standardization Initiative (NSI) alliance

**Affiliations:** 1 Department of Nephrology, Dialysis and Transplantation, International Renal Research Institute of Vicenza, San Bortolo Hospital, Viale Rodolfi 37, Vicenza, 36100 Italy; 2 Department of Management and Engineering, University of Padova, Vicenza, Italy; 3 Department of Health Sciences, Section of Anaesthesiology, Intensive Care and Pain, University of Florence, Florence, Italy; 4 Division of Critical Care Medicine, University of Alberta, Edmonton, AB Canada; 5 Department of Intensive Care, Austin Hospital, Department of Epidemiology and Preventive Medicine, Australian and New Zealand Intensive Care Research Centre, Monash University, Melbourne, VIC Australia; 6 Department of Medicine, Albany Medical College, Albany, NY 12209 USA; 7 Division of Intensive Care and Emergency Medicine, Department of Internal Medicine, Medical University of Innsbruck, Innsbruck, Austria; 8 Center for Critical Care Nephrology, Department of Critical Care Medicine, University of Pittsburgh, Pittsburgh, PA USA; 9 Department of Radiology and Biomedical Research Imaging Center, University of North Carolina at Chapel Hill, Chapel Hill, NC USA; 10 Division of Nephrology, University of California, San Diego, CA USA; 11 Department of Pediatric Cardiac Surgery, Bambino Gesù Children’s Hospital, Rome, Italy; 12 Department of Intensive Care, San Bortolo Hospital, Vicenza, Italy; 13 Purdue University College of Engineering, West Lafayette, IN USA; 14 Department of Intensive Care, Erasme Hospital, Université libre de Bruxelles, Brussels, Belgium

**Keywords:** Terminology, Diffusion, Convection, Ultrafiltration, Transmembrane pressure, CRRT membranes, CRRT modalities, Dose, CRRT efficiency, Clearance

## Abstract

This article reports the conclusions of a consensus expert conference on the basic principles and nomenclature of renal replacement therapy (RRT) currently utilized to manage acute kidney injury (AKI). This multidisciplinary consensus conference discusses common definitions, components, techniques, and operations of the machines and platforms used to deliver extracorporeal therapies, utilizing a “machine-centric” rather than a “patient-centric” approach. We provide a detailed description of the performance characteristics of membranes, filters, transmembrane transport of solutes and fluid, flows, and methods of measurement of delivered treatment, focusing on continuous renal replacement therapies (CRRT) which are utilized in the management of critically ill patients with AKI. This is a consensus report on nomenclature harmonization for principles of extracorporeal renal replacement therapies. Devices and operations are classified and defined in detail to serve as guidelines for future use of terminology in papers and research.

## Background

The management of critically ill patients with acute kidney injury (AKI) requiring renal replacement therapy (RRT) demands a multidisciplinary approach. In spite of previous efforts at harmonization, the terminology used to describe the different aspects and modalities of RRT is often confusing. A consensus conference on RRT terminology was organized to develop common definitions for the components, techniques, and operation of the machines and platforms used for acute extracorporeal therapies.

In this article, we report the conclusions of the consensus group on the basic principles underlying RRT technologies and the application of those principles to patient care, using “machine-centric” rather than “patient-centric” terminology. We provide a detailed description of the performance characteristics of membranes and filters, solute and fluid transport mechanisms across membranes, flow rate parameters, and methods of treatment evaluation, focusing on the continuous RRT (CRRT) used in the treatment of critically ill patients.

## Methodology

A conference was organized in Vicenza, Italy, to gather experts in CRRT and members of CRRT manufacturing companies to establish consensus on technical terminology and definitions relevant to basic principles of CRRT and related technology [1]. The conference provided the background for a modified Delphi consensus methodology as previously utilized for the Acute Disease Quality initiative consensus sessions [2]. Prior to the conference, participants screened the literature of the last 25 years and previous taxonomy efforts [3–5]. Keywords included “*continuous renal replacement therapy*”, “*dialysis*”, “*hemofiltration*”, “*convection*”, “*diffusion*”, “*ultrafiltration*”, “*dose*”, “*blood purification*”, “*renal support*”, “*multiorgan dysfunction*”, together with the relative MeSH terms. Abstracts of 707 articles were screened and more than 300 papers were read in full and analyzed. Based on this literature search, a series of definitions and terms were proposed and consensus was achieved from the majority of experts who participated in the conference. Where consensus was lacking, different statements were created after two-thirds of the audience expressed a positive vote. We present the results of this effort of terminology harmonization called NSI (Nomenclature Standardization Initiative).

## Characteristics of the membrane and filter

### Geometric characteristics

The main one-dimensional geometric characteristics of hollow fiber membranes are length (L), mean inner radius (r^−^_i_), wall thickness (t), and number of pores (N_p_). The membrane surface area depends on the number of fibers (N_f_). Using these parameters, multidimensional characteristics [6] can be expressed as listed in Table 1.

**Table 1:** Multidimensional characteristics of the membranes

Multidimensional characteristic	Symbol	Formula
Surface area	*A*	*A* = 2 ⋅ *N*_*f*_ ⋅ *L* ⋅ *π* ⋅ *r* ^−^_*i*_
Filter priming volume	*V*_*b*_^*F*^	*V*_*b*_^*F*^ = *N*_*f*_ ⋅ *L* ⋅ *π r* ^−^_*i*_^*2*^
Total priming volume	*V*_*b*_^*TOT*^	*V*_*b*_^*TOT*^ = *V*_*b*_^*F*^ *V*_*b*_^*TOT*^ + volume of tubes
Membrane porosity	*ρ*	*ρ* = *N*_*p*_ ⋅ *π* ⋅ *r* ^−^_*p*_^*2*^

*L* membrane length, *N*_*f*_ Number of fibers in the filter, *N*_*p*_ number of pores in the filter, *r* ^*–*^_*i*_ mean inner radius of the fibers, *r* ^*–*^_*p*_ mean inner radius of the pores

### Performance characteristics

The performance characteristics define the potential applications of each membrane.

#### Membrane ultrafiltration coefficient and filter ultrafiltration coefficient

The membrane ultrafiltration coefficient (K_UF_) represents the water permeability of the filter membrane per unit of pressure and surface. It depends on both the dimensions of the membrane and the number of pores and is measured as:

$$ {K}_{UF} = \frac{Q_{UF}}{TMP}\cdot \frac{1}{A} $$

where Q_UF_ is the ultrafiltration flow rate, TMP is the transmembrane pressure, and A is the membrane surface area. The unit of measurement is ml/h/mmHg/m^2^. Treatment parameters that enhance or reduce pore blockage induce changes in the K_UF_.

The filter ultrafiltration coefficient (DK_UF_) is defined as the product of the K_UF_ and membrane surface area (A):

$$ D{K}_{UF} = {K}_{UF}\cdot A $$

The unit of measurement is ml/h/mmHg. Membrane manufacturers measure DK_UF_ as the ratio of the Q_UF_ per unit of applied TMP.

The K_UF_ is used to define “high-flux” or “low-flux” membranes. Although there is no definitive consensus in the literature about the K_UF_ cut-off value [7], it is generally assumed that a K_UF_ <10 ml/h/mmHg/m^2^ identifies a low-flux membrane, a K_UF_ of 10–25 ml/h/mmHg/m^2^ identifies middle-flux membranes, and a K_UF_ >25 ml/h/mmHg/m^2^ identifies high-flux membranes.

The term high-flux has been generally used to define a membrane with an ultrafiltration coefficient >25 ml/h/mmHg/m^2^. This mainly describes the hydraulic permeability of the membrane (permeability to water). However, hydraulic permeability does not necessarily correspond to the permeability to solutes, which instead depends on the density of pores, the mean size of pores, and the distribution of pores. For this reason the terms high-flux and highly permeable membrane are not interchangeable.

#### Mass transfer area coefficient

The mass transfer area coefficient (K_0_A) represents the overall capacity of the membrane to provide diffusive removal of solutes over the entire filter surface. It is defined as the product of the solute flux per unit of membrane area (K_0_) and the membrane surface area. The unit of measurement is ml/min.

The K_0_A value can change during dialysis as a result of changes in membrane permeability or a loss of membrane exchange surface area.

#### Membrane sieving coefficient/rejection coefficient

The sieving coefficient (SC) is the ratio of a specific solute concentration in the ultrafiltrate (removed only by a convective mechanism), divided by the mean plasma concentration in the filter:

$$ SC=\frac{C_{UF}}{\left({C}_{Pi} + {C}_{Po}\right)/2} $$

where C_UF_ is the solute concentration in the ultrafiltrate, and C_Pi_ and C_Po_ the plasma solute concentrations at the inlet and outlet of the filter, respectively. A true calculation would require measurement of the solute concentration in plasma water rather than plasma to avoid interference of proteins. Nevertheless, for practical purposes, plasma concentration is normally accepted.

**Fig. 1:**
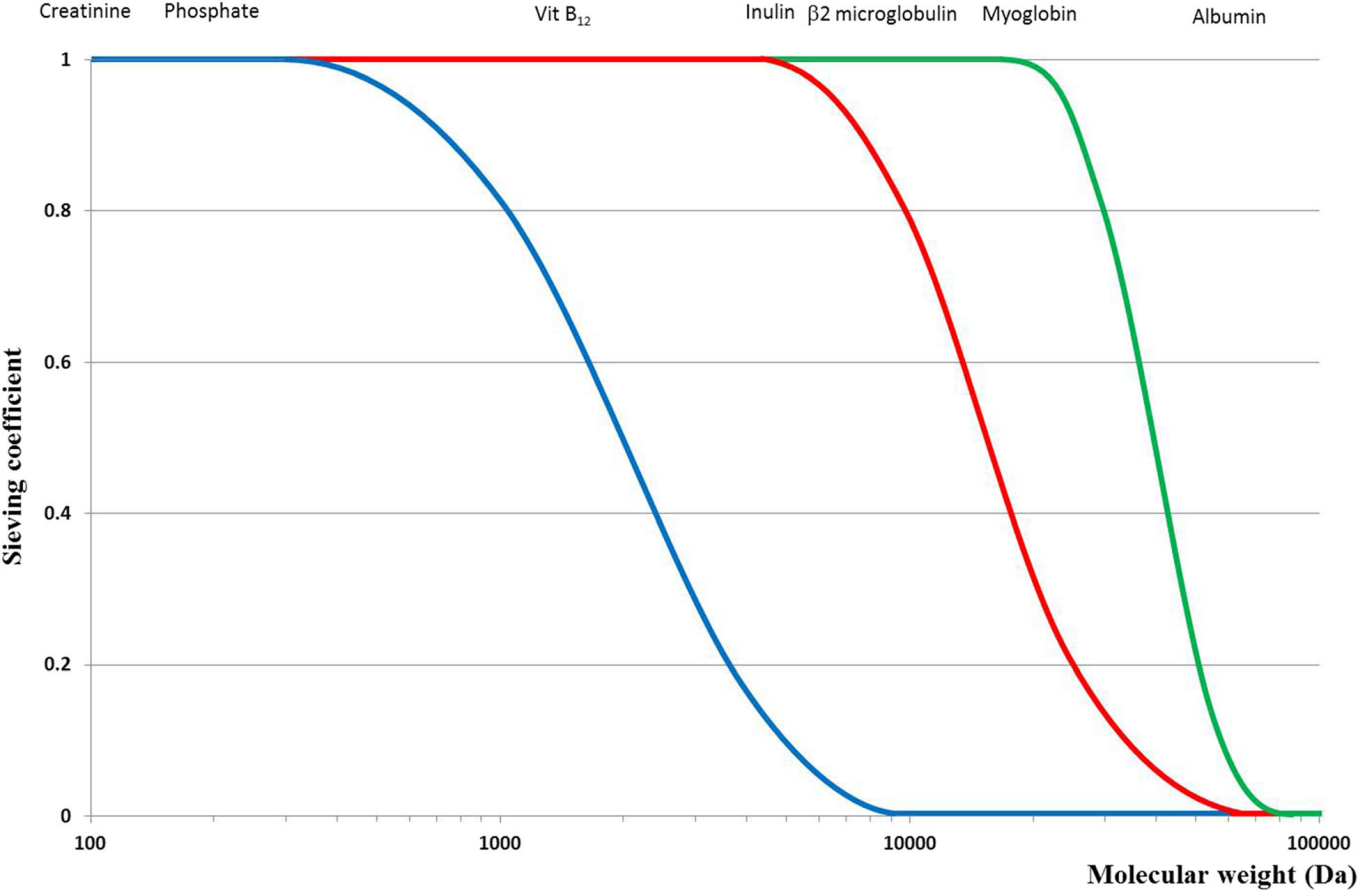
Schematic diagram of sieving coefficient profiles for low-flux (blue), high-flux (red) and high cut-off membranes (green)

SC is correctly measurable only in the absence of a gradient for diffusion (no concentration gradient through the membrane). Measurement of the SC varies during treatment because the characteristics of the membrane change. SC is specific for each solute and for every membrane (Fig. 1). The formula is commonly simplified to the ratio between the concentration in the ultrafiltrate and the concentration in pre-filter plasma.

The rejection coefficient (RC) is defined as:

$$ RC=1-SC $$

#### Cut-Off

For a specific membrane, the cut-off represents the molecular weight of the smallest solutes retained by the membrane. Taking into account the normal distribution of membrane pore size, the statistical cut-off value is identified as the molecular weight of a solute with a SC of 0.1. For a specific membrane, the retention onset (cut-off 90 % or 0.9) represents the molecular weight of a molecule with a SC of 0.9. For a complete understanding of the performance characteristics of a membrane, the cut-off value and the retention onset both need to be taken into account, allowing evaluation of the profile of the SC curve for each membrane (Fig. 1) [8].

Clinically, the expression “high cut-off membrane” describes membranes with a cut-off value that approximates the molecular weight of albumin (before exposure to blood or plasma).

## Mechanisms of solute and fluid transport

Solute transport occurs mainly by two phenomena: convection and diffusion. Fluid transport across semipermeable membranes is driven by ultrafiltration. Adsorption influences removal of hydrophobic (lipid-soluble) compounds by attachment of solute to the membrane. When solute removal rate (mass/time) is normalized by the concentration of blood/plasma entering the filter (mass/volume), the correct term to be used is “solute clearance” which is expressed in ml/min and describes the volume of blood completely purified by the solute in the unit of time.

### Ultrafiltration and convection

Ultrafiltration describes the transport of plasma water (solvent, free of cells and colloids) through a semipermeable membrane, driven by a pressure gradient between blood and dialysate/ultrafiltrate compartments. It is influenced by the intrinsic properties of the filter, such as the DK_UF_, and the operating parameters (e.g., TMP) [9]. Quantitatively, ultrafiltration is defined by the ultrafiltration rate (Q_UF)_:

$$ {Q}_{UF} = D{K}_{UF} \cdot \kern0.5em TMP $$

The term ultrafiltration requires some specifications depending on the context in which it is utilized. When ultrafiltration is applied to a circuit or a CRRT treatment, specifications should be made using terms such as total ultrafiltration (UF = overall ultrafiltration volume produced during treatment) and net ultrafiltration (UF^NET^ = net ultrafiltrate volume removed from the patient by the machine). In the first case, the overall volume can be completely replaced, partially replaced, or not replaced at all. UF^NET^ is the difference between UF and the volume replaced in the circuit (Table 2).

**Table 2:** Fluids and flows in continuous renal replacement therapy

Flowrate	Symbol	Unit of measure	Definitions and comments
Blood flowrate	Q_B_	ml/min	Depends on: - modality - vascular access - hemodynamic stability of the patient
Plasma flowrate	Q_P_	ml/min	Approximated as: Q_P_ = (1 – HCT) ∙ Q_B_ where HCT = hematocrit
Ultrafiltration flowrate	Q_UF_	ml/h	Total volume of fluid removed in the filter by positive TMP per unit of time: Q_UF_ = Q_UF_^NET^ + Q_R._ Depends on: - blood flow rate - filter and membrane design - transmembrane pressure (TMP) - membrane ultrafiltration coefficient and surface area
Net ultrafiltration flowrate (Δ weight flowrate) (weight loss flowrate)	Q_UF_^NET^	ml/h	Net volume of fluid removed from the patient by the machine per unit of time
Plasma ultrafiltration flow rate	Q_P-UF_	ml/h	Total volume of plasma removed in the plasma filter by TMP per unit of time
Replacement flowrate (Substitution flow rate) (Infusion flowrate)	Q_R_^PRE^ Q_R_^POST^ Q_R_^PRE/POST^	ml/h	Sterile fluid replacement can be: - upstream of filter (pre-replacement, pre-infusion or pre-dilution): reduced depurative efficiency but better filter life - downstream of filter (post-replacement, post-infusion or post-dilution): higher depurative efficiency but lower filter life - both upstream and downstream of filter (pre-post replacement, pre-post infusion or pre-post dilution): compromise between the two modalities
Replacement plasma flow rate	Q_P-R_	ml/h	Replacement of plasma downstream of the plasma filter
Dialysate flowrate	Q_D_	ml/h	Volume of dialysis fluid running into the circuit per unit of time
Effluent flowrate	Q_EFF_	ml/h	Waste fluid per unit of time coming from the outflow port of the dialysate/ultrafiltrate compartment of the filter: Q_EFF_ = Q_UF_ + Q_D_ = Q_UF_^NET^ + Q_R +_ Q_D_

When techniques are discussed, ultrafiltration may be isolated (no other mechanism is utilized in the treatment and only volume control is achieved), be used as part of hemofiltration (the ultrafiltrate is partially or completely replaced achieving volume and solute control), or combined with diffusion in treatments such as hemodialysis (HD) or hemodiafiltration (HDF). Different membranes are utilized for different techniques.

Convection is the process whereby solutes pass through membrane pores, dragged by fluid movement (ultrafiltration) caused by a hydrostatic and/or osmotic transmembrane pressure gradient.

The convective flux (J_c_) of a solute depends on the Q_UF_, the membrane surface area (A), the solute concentration in plasma (C_Pi_) and the solute SC:

$$ {J}_c = \frac{Q_{UF}}{A}\cdotp {C}_{Pi}\cdotp SC $$

Compared to diffusive transport, convective transport permits the removal of higher molecular weight solutes at a higher rate [10].

#### Transmembrane pressure

In hollow fiber filters, the TMP is the pressure gradient across the membrane. The terms that define this gradient are the hydrostatic pressure in the blood compartment (P_B_), the hydrostatic pressure in the dialysate/ultrafiltrate compartment (P_D_) and the blood oncotic pressure (π_B_). The TMP value varies with length (l) along the whole filter length (L):

$$ TMP(l)={P}_B(l)-{P}_D(l)-{\pi}_B(l) $$

Generally, TMP is expressed using a simplified formula:

$$ TM{P}^{*}=\frac{P_{Bi}+{P}_{Bo}}{2}-\frac{P_{Di}+{P}_{Do}}{2}-\frac{\pi_{Bi}+{\pi}_{Bo}}{2} $$

where P_Bi_ is the blood inlet pressure, P_Bo_ the blood outlet pressure, P_Di_ the dialysate/ultrafiltrate inlet pressure, P_Do_ the dialysate/ultrafiltrate outlet pressure, π_Bi_ the oncotic pressure of the inlet blood, and π_Bo_ the oncotic pressure of the outlet blood. It must be stressed that the TMP* is a positive, averaged value along the length of filter, and does not reflect the true local pressure profile in the filter. In other words, a positive TMP* does not imply a positive TMP (l) at each point in the filter.

Furthermore, CRRT machines do not usually directly measure the P_Di_ or the oncotic pressure, so the TMP is *estimated* using an even simpler formula:

$$ TM{P}^{*}=\frac{P_{PRE}+{P}_{OUT}}{2}-{P}_{EFF} $$

where P_PRE_ is the pre-filter pressure, P_OUT_ the post-filter pressure, and P_EFF_ the pressure measured in the effluent line (all three measured by the machine). In the most common configuration, as blood flows down the filter, plasma water is removed and eliminated with the spent dialysate (if present), which flows in a counter-current direction. This ultrafiltration, called direct (or internal) filtration, identifies the one-directional movement of plasma water from the blood side to the dialysate/ultrafiltrate compartment of the filter due to a local positive TMP(l):

$$ {P}_B(l)>{P}_D(l)+{\pi}_B(l) $$

At a critical point on the filter, where P_B_ (l) = P_D_ (l) + π_B_ (l), equilibrium is achieved. After this point, the TMP (l) may become negative (even if TMP* is positive) allowing dialysate fluid to flow back into the blood compartment, resulting in so-called back filtration [11]. Back filtration describes the movement of fluid from the dialysate compartment to the blood compartment.

### Diffusion

Diffusion is a process whereby molecules move randomly across a semipermeable membrane. Solute movement occurs from a more concentrated to a less concentrated area, until an equilibrium is reached between the two compartments. The concentration gradient (C_1_ – C_2_ = dc) is the driving force. The unidirectional solute diffusive flux (J_d_) through a semipermeable membrane follows Fick’s law of diffusion, being directly proportional to the diffusion coefficient (D) of the solute and inversely proportional to the distance between the compartments (dx) [10]:

$$ {J}_d\kern0.5em  = - D\kern0.5em \left(\frac{dc}{dx}\right) $$

The diffusivity coefficient D can be approximated using the Stokes-Einstein equation:

$$ D=\frac{k_BT}{6\pi \mu R} $$

where k_B_ is the Boltzmann constant, T the absolute temperature, μ the viscosity of the medium, and R the effective radius of the molecules. Assuming that most molecules are globular and their effective radius is proportional to the cube root of their molecular weight, D is higher for smaller molecular weight solutes [12].

### Adsorption

Adsorption is an extracorporeal process in which molecules dissolved in plasma or blood (in particular peptides and proteins) bind to the membrane structure or to other adsorbing substances such as charcoal, resins, or gels. The characteristics that influence molecule-membrane interaction are typical for each molecule (i.e., dimension, charge, and structure) and for each particular membrane (i.e., porosity, composition, hydrophobicity, surface potential). Adsorption cartridges should be evaluated in terms of their device adsorption capability (DAC) and their selectivity. DAC represents the total quantity of a specific molecule that the device is able to adsorb, and should be of the same order of magnitude as the blood concentration of that molecule multiplied by the blood volume. Selectivity is a safety parameter: it defines what the device does *not* adsorb.

## Modalities of extracorporeal RRT

### Hemodialysis

The main mechanism of solute removal in hemodialysis is diffusion, which is chiefly effective in the removal of small solutes. Hemodialysis involves the use of a *hemodialyzer*, where blood and dialysate solution circulate counter-current or co-current. A counter-current configuration is preferred because the average concentration gradient is kept higher along the whole length of the dialyzer. Conversely, a co-current configuration guarantees better stability and control of hydrodynamic conditions, and better air removal during the priming phase [13]. High-flux filters permit achievement of significant convective transport: this modality is called high-flux hemodialysis [14].

### Hemofiltration

Hemofiltration is an exclusively ultrafiltration/convection treatment in which high-flux membranes are utilized in the absence of dialysis fluid. Infusion of a sterile solution into the blood circuit reconstitutes the reduced plasma volume and reduces solute concentration. Infusion of a sterile solution (replacement fluid) can replace totally or partially the filtered volume. Replacement fluid can be infused pre-filter (*pre-dilution*) or post-filter (*post-dilution*). In terms of solute clearance, post-dilution is more efficient than pre-dilution, but can lead more easily to membrane fouling due to hemoconcentration [9].

### Hemodiafiltration

Hemodiafiltration combines hemodialysis and hemofiltration, whereby the mechanisms involved in solute removal are both diffusive and convective. Since this modality utilizes high-flux membranes, adequate amounts of sterile solution must be infused to replace the removed volume (pre-filter or post-filter) [15].

### Isolated ultrafiltration

The main goal of ultrafiltration is to remove fluid using semipermeable membranes without volume replacement, thus achieving volume but not solute control in the patient [16].

### Plasmapheresis

Membrane plasmapheresis filters the plasma through plasma filters and replaces it with plasma-derived products, such as fresh frozen plasma, albumin, or other fluids. Alternatively, plasma can be extracted gravimetrically from whole blood using a centrifuge pump. Plasmapheresis is used to remove hydrophilic and lipophilic high molecular weight pathogenic substances [17].

### Hemoperfusion/plasmaperfusion

In hemoperfusion or plasmaperfusion, blood or plasma circulates through a column containing specific sorbents, with adsorption as the only removal mechanism. Usually combined with other modalities, hemoperfusion and plasmaperfusion are used to remove specific hydrophobic (lipid-soluble) substances, toxins, or poisons [18].

### Fluids, volumes and flows

Solute transport during extracorporeal treatments strictly depends on the operating conditions including blood flow rate, dialysate, net ultrafiltration, and replacement flow rates, designed to achieve the desired clearance performance. These typical CRRT parameters (fluids and flows) are listed in Table 2.

#### Filtration fraction and concentration ratio

The filtration fraction (FF) is defined as the ratio between the ultrafiltration flow rate (Q_UF_) and the plasma flow rate (Q_P_):

$$ FF=\frac{Q_{UF}}{Q_P} $$

Filtration fraction can also be measured by the following equation:

$$ FF = \frac{1- Pro{t}_{IN}}{Pro{t}_{OUT}} $$

where Prot_IN_ is the protein concentration in plasma entering the filter and Prot_OUT_ is the protein concentration in plasma exiting the filter.

A directly measured FF can be expressed as a fraction:

$$ FF = \frac{Q_{UF}}{Q_P}=\frac{Q_{UF}}{Q_B\left(1-HCT\right)+{Q}_R^{PRE}} $$

where Q_R_^PRE^ is the pre-replacement flow rate and Q_B_ the blood flow rate.

For practical clinical purposes (as often used in CRRT machines) it is useful to define the concentration ratio (CR), which quantifies the magnitude of hemoconcentration inside the filter:

$$ CR=\frac{Q_{UF}}{Q_B + \kern0.5em {Q}_R^{PRE}}=\frac{Q_R^{POST} + {Q}_{UF}^{NET} + {Q}_R^{PRE}}{Q_B + \kern0.5em {Q}_R^{PRE}} $$

where Q_R_^POST^ is the post-replacement flow rate, Q_R_^PRE^ is the pre-replacement flow rate, and Q_UF_^NET^ the net ultrafiltration flow rate (all of which sum to *Q*_*UF*_). Clinically, while the filtration fraction should be kept ideally below 30 %, the CR should be kept below 20–25 % [19], depending on initial hematocrit, to reduce hemoconcentration and mitigate protein-membrane interactions.

### Treatment evaluation methods: the “dose” of RRT

Although the most appropriate dose has not been established for specific patients, large studies have demonstrated in the general population a direct relationship between dose and survival for both intermittent and CRRT modalities [20–26]. Today, a growing body of evidence suggests the use of precision CRRT, which is characterized by the need to pay great attention to the balance between demand (of blood purification) and capacity (of the native kidney). In these circumstances, personalized prescription and monitoring of treatment dose is highly recommended [27–30]. Although treatment adequacy should be considered more appropriately as a composite of different elements rather than an index based solely on urea kinetics, in CRRT a treatment efficiency equal or higher than 25 ml/kg/h is commonly considered adequate. This will approximately result in a daily standardized Kt/V = 1 which describes the efficacy of treatment for a specific patient.

Dose identifies the volume of blood cleared of waste products and toxins by the extracorporeal circuit per unit of time. In practice, it is measured as the rate of removal of a representative solute. Urea is the solute most commonly used to quantify dose [31] because it is an indicator of protein catabolism and is retained in kidney failure [12]. Originally, this solute-based approach was developed to measure the dose of dialysis prescribed to patients with end-stage renal disease. In these patients, application of this approach is relatively simple and correlates well with patient outcomes [20]. However, when using CRRT to treat critically ill patients, other measures of adequacy and dose should also be considered. One potentially easier and more reproducible means of estimating dose is incorporating the measurement of flow rates provided by the dialysis machine [32].

Multiple different definitions and formulas to calculate RRT “dose” have been proposed [33, 34]. In this section, we try to clarify the concept. During RRT, the definition of dose must include: target (patient), target (machine), current, average, projected, current effective delivered doses, and average effective delivered doses. Starting from these definitions, therapies should be identified by their efficiency, intensity, and efficacy.

#### Target dose (prescribed)

The target dose (prescribed) is the clearance prescribed for a specific patient in his/her specific clinical condition and represents the clearance the prescribing clinician wants to achieve in that patient.

#### Target machine dose (set)

The target machine dose is the clearance that the prescribing clinician wants to achieve from the machine. It is usually set as a target machine efficiency or by specifying the flow rate settings and RRT modality. The target machine dose can be modified during the treatment to reduce the mismatch between the target dose (prescribed) and the average effective delivered dose (measured).

#### Current dose (estimated from treatment parameters)

The current dose (estimated from treatment parameters) is the clearance at the present time estimated from the flow rates in the extracorporeal circuit. During downtime, when the machine treatment is stopped, the current dose is zero. Interruptions during the treatment can occur because of machine alarms, circuit clotting, vascular access malfunctions, or interruptions when the patient must leave the intensive care unit (ICU), such as for surgery or radiological investigations.

#### Average dose (measured/calculated)

The average dose is the clearance calculated for the current dose applied over the total treatment time. The total time of treatment is defined as the sum of the effective time of treatment and downtime. The effective time of treatment is the cumulative time during which the effluent pump is working. The average dose is usually an overestimate of the average effective delivered dose.

#### Projected dose (calculated/estimated)

The projected dose is the weighted-mean clearance that will theoretically be obtained at the end of the treatment. If the target machine dose is kept constant during treatment, the projected dose and the average dose will align. If the target machine dose is modified, the projected dose will depend on the average dose obtained until that moment and the new set target machine dose. The projected dose is usually an overestimate of the average effective delivered dose.

#### Current effective delivered dose (measured)

The current effective delivered dose (measured) is the clearance observed at every moment during the treatment. Unlike the current dose (estimated from treatment parameters), it is based on blood concentrations. The current effective delivered dose depends mainly on the specific RRT modality, treatment settings, and other technical and clinical issues that qualitatively and quantitatively affect clearance. The major determinants are differences between the displayed and real blood or effluent flow rates, inadequate vascular access, incorrect priming procedure, loss of surface area (clotting, air), loss of permeability (clotting of the membrane, protein cake deposition on the inner surface of membranes, concentration polarization), high blood viscosity and hematocrit, and excessive FF.

#### Average effective delivered dose (measured)

The average effective delivered dose (measured) or real dose is the clinically relevant (measured) clearance delivered to the patient. It is calculated on the basis of the weighted-mean of the current effective delivered dose, over the total time of treatment until that specific moment. The average effective delivered dose is the average of the current effective delivered dose during the time of treatment, and not of the current dose, because the latter is plagued by errors during times in which flow may be occurring with no solutes clearance, (e.g., bag changes, recirculation procedures). The largest discrepancies between the target dose and the average effective delivered dose are found in predominantly diffusion-based CRRT (i.e., continuous veno-venous hemodialysis and continuous veno-venous hemodiafiltration) [33].

In an ideal treatment, during which downtime and technical and/or clinical hindrances do not influence clearance, the target, target machine, current, average, projected, current effective delivered dose, and average effective delivered doses will be equal.

### Efficiency, intensity and efficacy

**Fig. 2:**
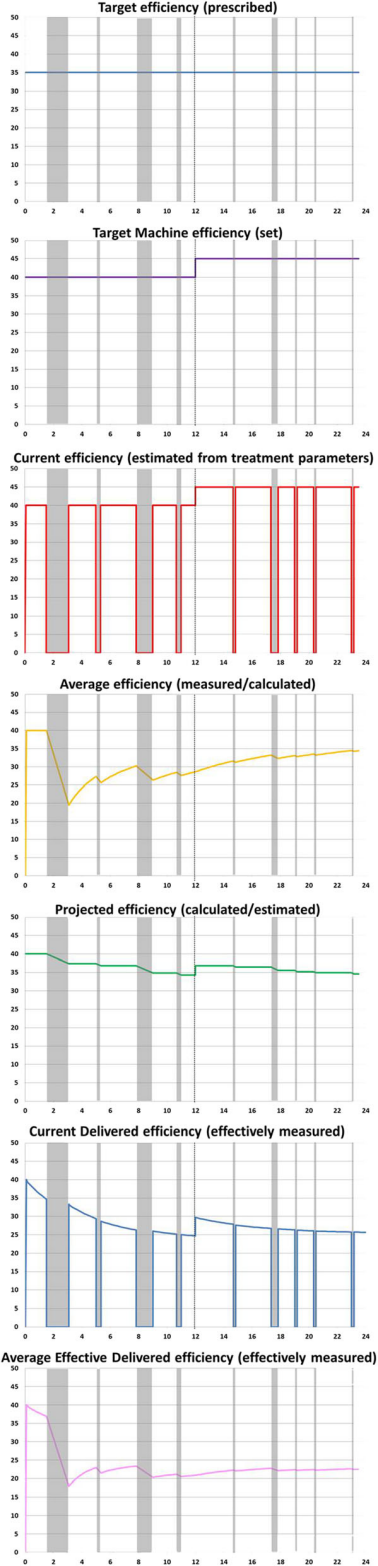
Practical example showing the different trends in efficiency (ml/kg/h, *y* axis) vs treatment time (h, *x* axis) during treatment with continuous renal replacement therapy (CRRT). *Target efficiency (prescribed)*: “It is the amount of clearance prescribed for the specific patient in his/her specific clinical condition, and represents the amount of clearance that the doctor wants to achieve in that patient. Example: according to literature, the doctor decides that a dose of 35 ml/kg/h is the most adequate for his patient”. *Target machine efficiency (set)*: “It is the amount of clearance that the physician wants to achieve in the machine. It is the only value that can be set in the machine. Example: taking into account the average downtime, the doctor sets the target machine dose to reach the target dose (prescribed). For example, to obtain a target dose (prescribed) of 35 ml/kg/h, the doctor sets flow rates and modalities to achieve a target machine dose of 40 ml/kg/h”. *Current dose (estimated from treatment parameters*): “It is the clearance at the present time, estimated considering the set flows in the extracorporeal circuit. During downtime, the current dose is zero. Example: based only on the instantaneous flow rates, the machine calculates the current dose at every moment of the treatment. A current dose of zero allows the user to recognize downtime”. *Average dose (measured/calculated)*: “It is the clearance calculated for the current dose applied over the total time of treatment. Example: based on the total time of treatment and the current dose calculated at every moment, the machine displays the average dose. At a particular moment of the treatment, if the average dose equals 35 ml/kg/h (the target dose prescribed), the physician can assume that the patient is undertreated”. *Projected dose (calculated/estimated)*: “It is the weighted-mean clearance that will theoretically be obtained at the end of the treatment. Example: based on the average dose obtained until a specific moment and the set target machine dose, the machine estimates the dose that theoretically will be obtained at the end of treatment session (24 h). At a particular moment during the treatment, if the projected dose is less than 35 ml/kg/h (target prescribed dose), the physician can assume that the patient will be undertreated at the end of the treatment”. *Current effective delivered dose (measured)*: “It is the amount of clearance observed at every moment during treatment time. Unlike the current dose, it is based on blood concentrations. Example: the doctor now calculates actual blood clearance based on concentrations of solute markers. He often finds differences with the current dose (estimated from treatment parameters) because technical issues in the measurement of flow rates limit the accuracy of the estimation”. *Average effective delivered dose (measured)*: “It is the clinically relevant amount of (measured) clearance delivered to the patient. It is calculated on the basis of the weighted-mean of the current effective delivered dose, over the total time of treatment until that specific moment”

Identified as a clearance (K), the efficiency represents the volume of blood cleared of a solute over a given period of time. It can be expressed as the ratio of blood volume over time (ml/min, ml/h, l/h, l/24 h, etc.) and is generally normalized to ideal patient weight (ml/kg/h). Efficiency depends on the reference molecules chosen (molecular size), removal mechanisms (diffusion, convection or both), and circuit operational characteristics (i.e., flow rates and type of filter). Efficiency can be used to compare different RRT treatments applied with the same modality using different settings and operational characteristics. Efficiency can be further categorized and defined as target efficiency, target machine efficiency, current efficiency, average efficiency, projected efficiency, current effective delivered efficiency, and average effective delivered efficiency. In Fig. 2, the different categories of efficiency during CRRT are illustrated with examples.

Intensity can be defined by the product “efficiency × time”. In practice, intensity represents the blood volume cleared of a solute after a certain period of time; it can be expressed as ml or l. When comparing RRT modalities with different duration times, the use of intensity is more appropriate than the use of efficiency. For example, despite its low efficiency, use of CRRT for a long period of time results in increased treatment intensity.

Renal failure patients frequently require more than a single treatment; therefore, frequency of treatment should be considered when assessing dose. Specifically, the product of intensity times frequency (measured as treatment days/week) is useful to obtain information beyond a single treatment. Although intensity allows comparison between different treatments, it does not take into account the volume of the solute pool.

Efficacy measures the removal of a specific solute achieved by a given treatment in a given patient. It can be identified as the ratio of the entire volume cleared during the treatment to the volume of distribution of that solute. In practice, efficacy is a dimensionless number and can be numerically defined as the ratio between intensity and the volume of distribution of a specific solute.

Definitions of efficiency, intensity, and efficacy, together with the related formulas and abbreviations, are given in Table 3.

**Table 3:** Definitions and formulas for efficiencies, intensities and efficacies

Measurement	Name	Symbol	Unit of measure	Formula
Efficiency	Target (prescribed)	*K*_*T*_	ml/kg/h	Assuming that the patient’s clinical condition does not change, K_T_ is a constant value throughout the treatment
Efficiency	Target machine	*K*_*Tm*_	ml/kg/h	Considering the downtime and the reduction in clearance properties of the membranes during treatment, *K*_*Tm*_ is usually set at a greater value than *K*_*T*_
Efficiency	Current	*K*_*Cr*_	ml/kg/h	${K}_{Cr}=\frac{\left({Q}_R^{PRE}+{Q}_D+{Q}_{UF}^{NET}+{Q}_R^{POST}\right)}{B.W.}\cdot \frac{Q_B\ }{Q_B + {Q}_R^{PRE}}$
Efficiency	Average	*K*_*Am*_	ml/kg/h	${K}_{Am}=\frac{1}{t_1}\cdot {\int}_0^{t_1} KCrdt$
Efficiency	Projected	*K*_*Pr*_	ml/kg/h	${K}_{Pr}=\frac{{\displaystyle {\int}_0^{t_1}}{K}_{Cr}dt + \left({t}_{tot}-{t}_1\right) \cdot {K}_{Tm}^{\hbox{'}}}{t_{tot}}$ where *K*_*Tm*_^'^ is the new target machine efficiency set
Efficiency	Current effective delivered	*K*_*Cd*_	ml/kg/h	${K}_{Cd}=\left({Q}_B\cdot \frac{C_{Bi}-{C}_{Bo}}{C_{Bi}}+{Q}_{UF}\cdot \frac{C_{Bo}}{C_{Bi}}\right)\cdot \frac{1}{B.W.}$
Efficiency	Average effective delivered	*K*_*Aed*_	ml/kg/h	${K}_{Aed}=\frac{1}{t_1}\cdot {\displaystyle \underset{0}{\overset{t_1}{\int }}}{K}_{Cd}dt$
Intensity	Target (prescribed)	*I*_*T*_	ml/kg	Blood volume that should be cleared applying *K*_*T*_ during the total time of treatment
Intensity	Target machine	*I*_*Tm*_	ml/kg	Blood volume that should be cleared applying *K*_*Tm*_ during the total time of treatment
Intensity	Current	*I*_*Cr*_	ml/kg	*I*_*Cr*_ = *K*_*Cr*_ ⋅ *t*_*tot*_
Intensity	Average	*I*_*Am*_	ml/kg	${I}_{Am}\kern0.5em =\kern0.5em {K}_{Cm}\cdot {t}_1\kern0.5em =\kern0.5em {\int}_0^{t_1}{K}_{Cr}dt$
Intensity	Projected	*I*_*Pr*_	ml/kg	${I}_{Pr}={K}_{Pr}\cdot {t}_{tot}=\kern0.5em {\int}_0^{t_1}{K}_{Cr}dt + \left({t}_{tot}-{t}_1\right) \cdot {K}_{Tm}^{\hbox{'}}$
Intensity	Current effective delivered	*I*_*Cd*_	ml/kg	*I*_*Cd*_ = *K*_*Cd*_ ⋅ *t*_1_
Intensity	Average effective delivered	*I*_*Aed*_	ml/kg	${I}_{Aed}={K}_{Ced}\cdot {t}_1\kern0.5em =\kern0.5em {\int}_0^{t_1}{K}_{Cd}dt$
Efficacy	Target (prescribed)	*E*_*T*_	Dimensionless	Solute removal obtained applying *I*_*T*_ to the volume of distribution of the solute
Efficacy	Target machine	*E*_*Tm*_	Dimensionless	Solute removal obtained applying *I*_*Tm*_ to the volume of distribution of the solute
Efficacy	Current	*E*_*Cr*_	Dimensionless	${E}_{Cr}=\frac{I_{Cr}}{V}=\frac{K_{Cr} \cdot {t}_{tot}}{V}$
Efficacy	Average	*E*_*Am*_	Dimensionless	${E}_{Am}=\frac{I_{Cm}}{V}=\frac{1}{V}{\int}_0^{t_1}{K}_{Cr}dt$
Efficacy	Projected	*E*_*Pr*_	Dimensionless	${E}_{Pr}=\frac{I_{Pr}}{V}=\frac{1}{V}\cdot \left[{\int}_0^{t_1}{K}_{Cr}dt + \left({t}_{tot}-{t}_1\right) \cdot {K}_{Tm}^{\hbox{'}}\right]$
Efficacy	Current effective delivered	*E*_*Cd*_	Dimensionless	${E}_{Cd}=\frac{I_{Cd}}{V}=\frac{K_{Cd}\cdot {t}_1}{V}=\frac{1}{V}\cdot \left({Q}_B\cdot \frac{C_{Bi}-{C}_{Bo}}{C_{Bi}}+{Q}_{UF}\cdot \frac{C_{Bo}}{C_{Bi}}\right)\cdot \frac{1}{B.W.} \cdot {t}_1$
Efficacy	Average effective delivered	*E*_*Aed*_	Dimensionless	${E}_{Aed}=\frac{I_{Ced}}{V}=\frac{K_{Ced}\cdot {t}_1}{V}=\frac{1}{V}\cdot {\int}_0^{t_1}{K}_{Cd}dt$

*B.W*. ideal body weight, *C*_*Bi*_ pre-filter blood concentration of the reference solute, *C*_*BO*_ post-filter blood concentration of the reference solute, *dt* delta time, *Q*_*B*_ blood flow rate, *Q*_*D*_ dialysate flow rate, *Q*_*R*_^*POST*^ post-replacement flow rate, *Q*_*R*_^*PRE*^ pre-replacement flow rate, *Q*_*UF*_^*NET*^ net ultrafiltration flow rate, *Q*_*UF*_ ultrafiltration flow rate, *t*_*tot*_ total time of treatment, *V* volume of distribution of the reference solute

## Conclusions

Understanding the basic mechanisms underlying the process of RRT is essential to be able to make appropriate treatment choices for individual patients. Although apparently simple, those choices are in reality complex, and specific to each clinical situation.

The aim of this consensus is to standardize the nomenclature used by all parties involved in planning and delivering RRT at any level. We hope that the industry will also adopt this standard terminology in the future.

## Abbreviations

r^−^_i_: Mean inner radius of the fibers
r^−^_p_: Mean inner radius of the membrane pores
*ρ*: Membrane porosity
*π*_*B*_: Oncotic pressure in the blood
*π*_*Bi*_: Oncotic pressure of blood inlet
*π*_*Bo*_: Oncotic pressure of blood outlet
A: Membrane surface area
AKI: Acute kidney injury
B.W.: Ideal body weight
C_Bi_: Pre-filter blood concentration of the reference solute
C_Bo_: Post-filter blood concentration of the reference solute
C_Pi_: Pre-filter plasma concentration of the reference solute immediately before the filter
C_Po_: Post-filter plasma concentration of the reference solute immediately after the filter
CR: Concentration ratio
CRRT: Continuous renal replacement therapy
C_UF_: Concentration of the reference solute in the ultrafiltrate
D: Diffusion coefficient
DAC: Device adsorption capability
dc: Concentration gradient
DK_UF_: Filter ultrafiltration coefficient
dx: Distance between compartments
E_Aed_: Average effective delivered efficacy
E_Am_: Average efficacy
E_Cd_: Current effective delivered efficacy
E_Cr_: Current efficacy
E_Pr_: Projected efficacy
E_T_: Target efficacy
E_Tm_: Target machine efficacy
FF: Filtration fraction
HCT: Hematocrit
I_Aed_: Average effective delivered intensity
I_Am_: Average intensity
I_Cd_: Current effective delivered intensity
I_Cr_: Current intensity
ICU: Intensive care unit
I_Pr_: Projected intensity
I_T_: Target intensity
I_Tm_: Target machine intensity
J_c_: Convective flux
J_d_: Diffusive flux
K: Clearance
K_0_: Solute flux per unit of membrane area
K_0_A: Mass transfer area coefficient
K_Aed_: Average effective delivered efficiency
K_Am_: Average efficiency
k_B_: Boltzmann constant
K_Cd_: Current effective delivered efficiency
K_Cr_: Current efficiency
K_Pr_: Projected efficiency
K_T_: Target efficiency (prescribed)
K'_Tm_: New target machine efficiency set at time t_1_
K_Tm_: Target machine efficiency
K_UF_: Membrane ultrafiltration coefficient
l: Infinitesimal part of the membrane’s length
L: Length of the membrane
N_f_: Number of fibers in the filter
N_p_: Number of pores in the membrane of the filter
P_B_: Hydrostatic pressure in the blood compartment
P_Bi_: Hydrostatic pressure in the inlet part of the blood compartment
P_Bo_: Hydrostatic pressure in the outlet part of the blood compartment
P_D_: Hydrostatic pressure in the dialysate/ultrafiltrate compartment
P_Di_: Hydrostatic pressure in the inlet part of the dialysate compartment
P_Do_: Hydrostatic pressure in the outlet part of the dialysate compartment
P_EFF_: Pressure in the effluent line
P_OUT_: Pressure in the out-flow line
P_PRE_: Blood pre-filter pressure measured by the machine
Prot_OUT_: Protein concentration in plasma exiting the filter
Prot_IN_: Protein concentration in plasma entering the filter
Q_B_: Blood flow rate
Q_D_: Dialysate flow rate
Q_EFF_: Effluent flow rate
Q_P_: Plasma flow rate
Q_P-R_: Replacement plasma flow rate
Q_P-UF_: Plasma ultrafiltration flow rate
Q_R_: Total replacement flow rate
Q_R_^POST^: Replacement flow rate post-filter
Q_R_^PRE^: Replacement flow rate pre-filter
Q_UF_: Ultrafiltration flow rate
Q_UF_^NET^: Net ultrafiltration flow rate
R: Radius of the molecules
RC: Rejection coefficient
RRT: Renal replacement therapy
SC: Sieving coefficient
T: Absolute temperature
t: Thickness of the fibers
t_1_: Treatment elapsed time (0 < t_1_ < t_tot_)
TMP*: Approximated cumulative pressure gradient across the entire membrane
TMP: Transmembrane pressure
t_tot_: Total time of treatment
UF: Overall ultrafiltration volume produced during treatment
UF^NET^: Net ultrafiltrate volume removed from the patient by the machine
V: Volume of distribution of the reference solute
V_b_^F^: Filter priming volume
V_b_^TOT^: Total priming volume
μ: Viscosity

## References

[1] Ronco C (2015). The Charta of Vicenza. Blood Purif.

[2] Kellum JA, Bellomo R, Ronco C (2008). Acute Dialysis Quality Initiative (ADQI): methodology. Int J Artif Organs.

[3] Ronco C, Bellomo R (1995). Continuous renal replacement therapies: the need for a standard nomenclature. Contrib Nephrol.

[4] Ronco C, Bellomo R (1998). Continuous renal replacement therapy: evolution in technology and current nomenclature. Kidney Int Suppl.

[5] Cerda J, Ronco C (2009). Modalities of continuous renal replacement therapy: technical and clinical considerations. Semin Dial.

[6] Clark WR, Hamburger RJ, Lysaght MJ (1999). Effect of membrane composition and structure on solute removal and biocompatibility in hemodialysis. Kidney Int.

[7] Uhlenbusch-Korwer I, Bonnie-Schorn E, Grassman A, Vienken J. Performance parameters. In: Vienken J, editors. Understanding Membranes and Dialyzers, vol 5. Pabst. 2004. p. 103–55.

[8] Boschetti-de-Fierro A, Voigt M, Storr M, Krause B (2013). Extended characterization of a new class of membranes for blood purification: the high cut-off membranes. Int J Artif Organs.

[9] Ficheux A, Ronco C, Brunet P, Argiles A. The ultrafiltration coefficient: this old 'grand inconnu' in dialysis. Nephrol Dial Transplant. 2015;30(2):204-8. 10.1093/ndt/gft493 PMC4309188 24362905

[10] Ronco C, Ghezzi PM, Brendolan A, Crepaldi C, La Greca G (1998). The haemodialysis system: basic mechanisms of water and solute transport in extracorporeal renal replacement therapies. Nephrol Dial Transplant.

[11] Rangel AV, Kim JC, Kaushik M, Garzotto F, Neri M, Cruz DN, Ronco C (2011). Backfiltration: past, present and future. Contrib Nephrol.

[12] Leonard E. The bases of Mass Separation Processes. In: Ronco C, Bellomo R, Kellum J, editors. Critical Care Neprology. Saunders Elsevier; 2009. p. 1131-5.

[13] Kim JC, Cruz D, Garzotto F, Kaushik M, Teixeria C, Baldwin M, Baldwin I, Nalesso F, Kim JH, Kang E (2013). Effects of dialysate flow configurations in continuous renal replacement therapy on solute removal: computational modeling. Blood Purif.

[14] Lee K, Jeong JH, Mun CH, Lee SR, Yoo KJ, Park YW, Won YS, Min BG (2007). Convection-enhanced high-flux hemodialysis. Artif Organs.

[15] Ronco C (2007). Evolution of hemodiafiltration. Contrib Nephrol.

[16] Costanzo MR, Ronco C (2012). Isolated ultrafiltration in heart failure patients. Curr Cardiol Rep.

[17] Nakanishi T, Suzuki N, Kuragano T, Nagasawa Y, Hasuike Y (2014). Current topics in therapeutic plasmapheresis. Clin Exp Nephrol.

[18] Winchester JF (2002). Sorbent hemoperfusion in end-stage renal disease: an in-depth review. Adv Ren Replace Ther.

[19] Ledebo I (1998). Principles and practice of hemofiltration and hemodiafiltration. Artif Organs.

[20] Eknoyan G, Beck GJ, Cheung AK, Daugirdas JT, Greene T, Kusek JW, Allon M, Bailey J, Delmez JA, Depner TA (2002). Effect of dialysis dose and membrane flux in maintenance hemodialysis. N Engl J Med.

[21] Ronco C, Bellomo R, Homel P, Brendolan A, Dan M, Piccinni P, La Greca G (2000). Effects of different doses in continuous veno-venous haemofiltration on outcomes of acute renal failure: a prospective randomised trial. Lancet.

[22] Ricci Z, Ronco C (2005). Renal replacement II: dialysis dose. Crit Care Clin.

[23] Ronco C, Cruz D, Oudemans van Straaten H, Honore P, House A, Bin D, Gibney N (2008). Dialysis dose in acute kidney injury: no time for therapeutic nihilism—a critical appraisal of the Acute Renal Failure Trial Network study. Crit Care.

[24] Bellomo R, Cass A, Cole L, Finfer S, Gallagher M, Lo S, McArthur C, McGuinness S, Myburgh J, Norton R (2009). Intensity of continuous renal-replacement therapy in critically ill patients. N Engl J Med.

[25] Palevsky PM, Zhang JH, O'Connor TZ, Chertow GM, Crowley ST, Choudhury D, Finkel K, Kellum JA, Paganini E, Schein RM (2008). Intensity of renal support in critically ill patients with acute kidney injury. N Engl J Med.

[26] Schiffl H, Lang SM, Fischer R (2002). Daily hemodialysis and the outcome of acute renal failure. N Engl J Med.

[27] Ostermann M, Joannidis M, Pani A, Floris M, De Rosa S, Kellum JA, Ronco C (2016). Patient Selection and Timing of Continuous Renal Replacement Therapy. Blood Purif.

[28] Bagshaw SM, Chakravarthi MR, Ricci Z, Tolwani A, Neri M, De Rosa S, Kellum JA, Ronco C (2016). Precision Continuous Renal Replacement Therapy and Solute Control. Blood Purif.

[29] Cerda J, Baldwin I, Honore PM, Villa G, Kellum JA, Ronco C (2016). Role of Technology for the Management of AKI in Critically Ill Patients: From Adoptive Technology to Precision Continuous Renal Replacement Therapy. Blood Purif.

[30] Murugan R, Hoste E, Mehta RL, Samoni S, Ding X, Rosner MH, Kellum JA, Ronco C (2016). Precision Fluid Management in Continuous Renal Replacement Therapy. Blood Purif.

[31] Garred L, Leblanc M, Canaud B (1997). Urea kinetic modeling for CRRT. Am J Kidney Dis.

[32] Ricci Z, Bellomo R, Ronco C (2006). Dose of dialysis in acute renal failure. Clin J Am Soc Nephrol.

[33] Lyndon WD, Wille KM, Tolwani AJ (2012). Solute clearance in CRRT: prescribed dose versus actual delivered dose. Nephrol Dial Transplant.

[34] Clark WR, Turk JE, Kraus MA, Gao D (2003). Dose determinants in continuous renal replacement therapy. Artif Organs.

